# Development of flexible durable multi-slotted antenna for wearable applications

**DOI:** 10.1016/j.heliyon.2024.e40627

**Published:** 2024-11-21

**Authors:** Kummaramsetty Sainath, Shine Let Gunamony, Wahaj Abbas Awan, Navin M. George, Majjiga Deva Sindhu, Fahad N. Alsunaydih, Khaled Alhassoon

**Affiliations:** a Department of ECE, Karunya Institute of Technology and Sciences, Coimbatore, India; b Department of Information and Communication Engineering, Chungbuk National University, Cheongju, 28644, South Korea; c Department of Electrical Engineering, College of Engineering, Qassim University, Buraydah, 52571, Saudi Arabia

**Keywords:** Flexible electronics, Slotted antenna, Silicone rubber, Specific absorption rate, Wearable IoT applications

## Abstract

In this study, a multi-slotted antenna is designed and characterized that can be used for wearable applications by utilizing a flexible, durable silicone rubber substrate. Flexible material has become increasingly popular among researchers in recent years for the development of wearable antennas for body area networks (BAN). The flexible device should be small in size so that it can be easily worn on the human body by integrating with wearables for transmitting and receiving signals over a sufficiently long distance. Unlike conventional flexible and semi-flexible substrate, presented work utilize silicon rubber substrate owing the advantages of good thermal stability and good resistance in varying environmental conditions. The designed antenna's surface offers a compact size of 14 × 12 mm^2^ with a thickness of 2 mm. The resonance frequency of the designed antenna is optimized at 5.8 GHz with a peak gain of 2.46 dBi. The specific absorption rate (SAR) achieved for the proposed antenna is 1.56 W/kg for 1 g of tissue which fulfills the FCC standard. The design offers radiation efficiency of around 80 % when loaded with hand model over the operational band of 5.5 GHz–6.5 GHz. The bending characteristics of multi-slotted antenna are also analyzed, which offers stable performance as compared to antenna without any bend. Compactness as well as high radiation efficiency have been accomplished that makes the proposed antenna a strong candidate for military applications, medical applications, GPS, RFID, and fitness tracking.

## 1 Introduction

The necessity for advanced communication systems has increased dramatically as wearable technology continues to advance quickly, urging new advancements in antenna design [1,2]. As the worlds of wearables and communication technologies intertwine, there is an increasing demand for antennas that seamlessly blend into wearables including clothing, ensuring uninterrupted connectivity while also prioritizing user comfort and durability [3,4]. As wearable technology integrates further into our daily routines, the limitations of traditional antennas become increasingly apparent [5]. Overcoming obstacles such as consistent communication, physical flexibility, and long-lasting durability in the realm of wearables calls for a revolutionary approach to antenna construction [[6], [7], [8]].

Today, the majority of academics and researchers are focusing on the design of flexible devices such as soft grippers, meta-absorbers, and frequency selective surfaces for a variety of applications [[9], [10], [11]]. Flexible antennas also grab a lot attention, being a key part of communication using flexible electronic systems. Characteristics such as being easily bent, wrinkled, stressed, or collapsed are a few prevalent qualities of flexible rf components in general and antennas as specific components [12,13]. By integrating the RF front end and the radiating parts on the same substrate, the textile-based antenna can also create a small and affordable feed network. The entire manufacturing cost of a microstrip wearable antenna is reduced when the radiating elements are incorporated on the same substrate [14,15]. In the past few years, numerous authors have discussed flexible Microstrip antennas in their research papers regarding wearable telemedicine applications. Diverse flexible dielectric substrates profound in the antenna design literature are polyamide, silicon rubber, polydimethylsiloxane (PDMS), neoprene rubber, polyethylene, Rogers RO3006, cloth materials, polycarbonate, etc [[16], [17], [18]]. Among these material silicon rubber substrates commonly called elastomer, or rubber-like material, made of a polymer that contains silicon along with carbon, oxygen, and hydrogen [19] offers various advantages not limited to low relative permittivity with highly inert which help them to not react with most of the substances. Moreover, to enhance the quality and lower the cost, some rubbers incorporate fillers [20]. Silicone rubber substrate is a low-cost, flexible, durable, easily available material.

The authors in Ref. [21] preferred a Rogers RO3006 substrate and a concentric rectangular split ring resonator metamaterial structure is introduced in the antenna design to decrease the SAR for wireless mobile applications. A denim flexible substrate is used by the authors in Ref. [22], and a circular edge-slotted radiating structure is proposed. For intrabody telemetry applications, a flexible antenna is proposed by considering photo paper as a substrate. For wireless body area networking (WBAN), telemetry, and medical wireless applications, the ISM band 2.45 GHz or 5.8 GHz resonant frequency is used [23]. In Ref. [24], the rubber material is used as the substrate for the flexible antenna design at 1.4 GHz. A flexible silicon rubber substrate with a concentric circle radiating patch is proposed for telemetry applications at 2.45 GHz [25]. A biocompatible polyethylene naphthalate substrate is also considered for flexible antenna configuration [26]. Rogers Duroid RO3003TM, a semi-flexible substrate is also considered for wearable application antenna design in Ref. [27]. Cancer tissue detection is investigated by having a flexible UWB antenna embedded inside a finger/breast in Ref. [28]. A dual-band multifunctional body-worn textile antenna is proposed in Ref. [29]. The antenna concept is first proposed with a single feeding port design, which achieves a frequency tuning range of 60 % in the lower band. This second antenna is optimized to have an isolation of at least 15 dB between its two ports for operation with two co-existing transceiver systems. Other flexible substrate materials, including PDMS, Teflon, Polyethylene, Polyamide, RT Duroid, and Epoxy, are used in the design of the rectangular microstrip patch antenna (RMSA) to regulate the 2.4–2.6 GHz operating frequency [30].

Apart from wearable applications, flexible antenna designs are suggested for 5G wireless communication, wireless power transfer, and millimeter-wave applications in order to integrate with the upcoming flexible electronics. For 5G sub-6-GHz communication systems, a wide-band ROGERS RO4835T-based flexible antenna is suggested in Ref. [31]. To prevent interference issues in the frequency range, hexagonal split-ring resonators are preferred as the radiating element provides broadband functionality for sub-6 GHz. These resonators can be separately delimited without compromising antenna performance. For RFID tagged systems, a multiband functioning flexible transparent antenna design is presented by the authors in Ref. [32]. This design provides wireless power transfer demonstration in near-field and far-field scenarios at 13.56 MHz and 2.45 GHz resonant frequencies respectively. A flexible antenna is presented in Ref. [33] comprising a rectangular patch embedded in a flexible PDMS substrate. The antenna's longevity and stability will be increased by the flexible PDMS substrate's strong tensile strength, low permeability to oxygen, and great thermal stability. Similarly, in Ref. [34], PDMS-based UWB flexible design is presented and the characteristics of UWB are examined at seven different resonant frequencies including the ISM bands. The antennas are small-sized, made of flexible material, operating at the whole UWB band as well as in the ISM 2.4 GHz band, thus can be used in portable telemedicine applications. A compact quad-band antenna proposed for automotive applications is discussed in Ref. [35]. A liquid crystal polymer substrate is used as it has good mechanical and resistive properties against bending and is very cost-efficient. Teflon is a particularly advantageous material for wearable antennas due to its low-loss tangent, which leads to increased radiation and minimal signal loss, even at high frequencies as discussed in Ref. [36]. Moreover, a number of researchers utilize other flexible materials like thin-film non-fabric, fabric, silicon-based materials, and Rogers RT5880 to achieve flexibility in antenna designs [[37], [38], [39]]. Likewise, to achieve compactness in the design, the structure of the radiating patch and ground plane are well tuned to achieve a resonance on either 2.45 GHz or 5.8 GHz ISM band, used commonly for wearable, medical body-worn applications [40,41].

Unlike the typical substrate, this paper utilizes a silicone rubber as substrate to design a multi-slotted antenna for flexible devices targeting on-body communication. Silicone rubber substrates are selected owing to the advantage of high flexibility while using thicker sizes which results in high durability. The proposed antenna is achieved by modifying rectangular printed antenna by etching horizontal as well as vertical stubs. Conformability analyses are carried out along with SAR study to verify the potential for flexible and WBAN devices. The paper is divided into the following section to better explain the various steps followed to design and characterize the proposed antenna. Section 2 provides the antenna design steps in detail. The designed multi-slotted antenna simulated and measured results are discussed in section 3. Section 4 details the bending analysis of the suggested flexible antenna. The performance of the multi-slotted antenna on the User's left hand is elaborated in section 5. Section 6 concludes the paper.

## 2 Multi-slotted wearable antenna design

The integration of multi-slots etched from the radiating patch has become a key strategy in developing miniaturized antennas adapted for wearable applications. From the literature insights [42,43], the multi-slotted antenna structure is suggested across diverse fields such as breast cancer detection, IoT applications, and 5G millimeter wave applications. The multi-slotted design approach facilitates bandwidth enhancement, reduction in the overall antenna size, and favorable radiation behavior. Furthermore, the capability of frequency tuning by positioning/adjusting the dimensions of the slots optimizes the antenna performance for specific frequency bands and communication standards.

### 2.1 Proposed antenna design

**Fig. 1:**
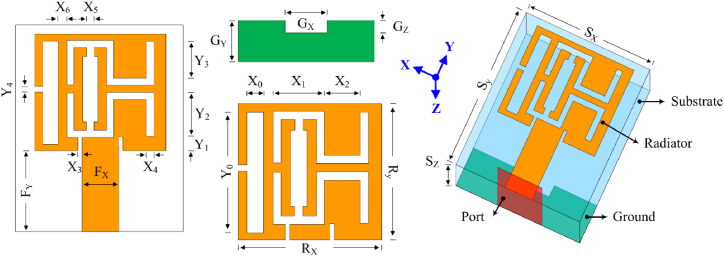
Proposed antenna design along with simulation setup.

The geometrical configuration of proposed rubber based compact conformable antenna is depicted in Fig. 1. Radiator of the proposed work is precisely designed by etching numerous rectangular slots along with a defected ground structure resulting in compact sized antenna while maintaining high performance. A 2 mm thick silicone-based rubber with highly comfortable properties and durable properties is utilized as a substrate of the antenna having a permittivity of 2.89, and a loss tangent of 0.02. The radiator is extracted from conventional rectangular patch shape by etching slots to lower the operating frequency of the antenna while a rectangular slot is etched from the partial ground plane, present at the back of the substrate, to improve the impedance matching at the resonating frequency. Both multi-slotted radiating patch and defected ground structure (DGS) are made up of flexible copper sheet having a thickness of 0.035 mm. The shape of the antenna is etched from a sheet using computer numerical control (CNC) machine cutting with high precision. Afterwards, fabric glue is utilized to join the radiating structure and DGS with the flexible silicon rubber substrate. Below are the optimized value of various parameters of proposed antenna presented in Fig. 1.

$$S_{X}=12;\;S_{Y}=14;\;S_{Z}=2;\;G_{X}=2;\;G_{Y}=5;\;G_{Z}=0.2;\;R_{X}=R_{Y}=8;\;F_{X}=2;\;F_{Y}=5;\;X_{0}=1;\;X_{1}=3;\;X_{2}=1.2;\;X_{3}=0.3;\;X_{4}=0.5;\;X_{5}=0.4;\;X_{6}=0.5;\;Y_{0}=7;\;Y_{1}=1;\;Y2=3;\;Y_{3}=2.5;\;Y_{4}=0.4.\;\left(\text{unit}\;\text{is}\;\text{mm}\right)$$

### 2.2 Antenna design methodology

**Fig. 2:**
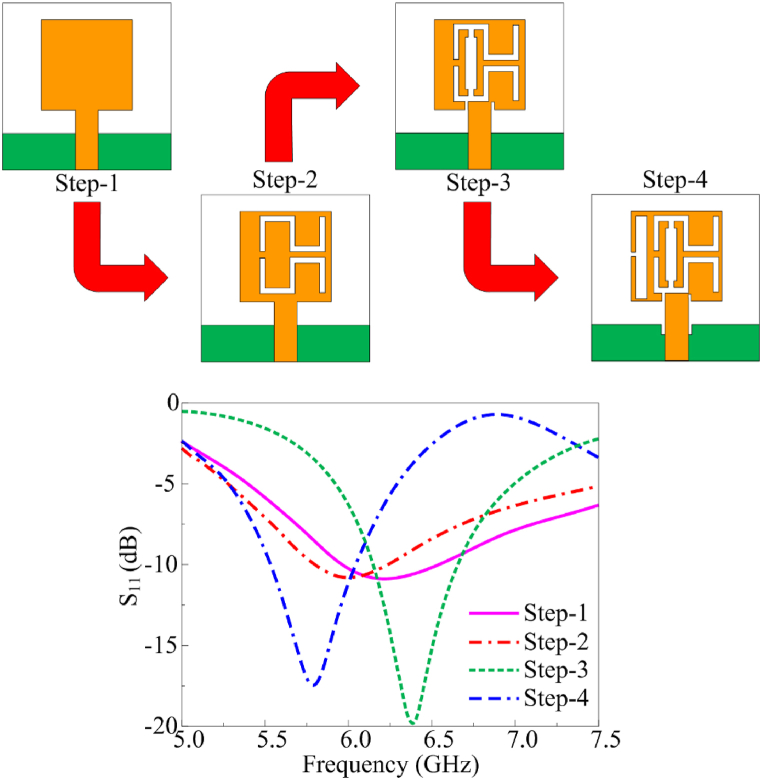
Antenna design methodology along with respective return loss results.

Fig. 2 depicts the stepwise evolution of the multi-slotted wearable antenna. As stated earlier, the antenna design methodology is initiated with the design of rectangular shaped monopole antenna. The antenna (step-1) operates at 6.4 GHz with impedance bandwidth of 6.21–6.74 GHz, as shown in Fig. 2. Afterwards a pair of meandered line shaped slots are etched from the radiator (Step-2) to lower the operating frequency. Although the resonating frequency is shifted around 6.25 GHz, the return loss become significantly high. Therefore, further slots are introduced to improve the impedance matching along with shifting the resonating frequency towards the lower side. Antenna in step-3 results in resonance around 6 GHz, however, the impedance matching remains still poor, as depicted in Fig. 2. Finally, a slot is etched in the ground that consequently improves the matching, along with that, the meandered line structure is further tunned to achieve the resonance of 5.8 GHz, as illustrated in Fig. 2. The optimized antenna offers S_11_ > −10 dB impedance bandwidth ranges from 5.62 GHz to 6.16 GHz, covering globally allocated ISM band spectrum.

### 2.3 Proposed antenna circuit modelling

**Fig. 3:**
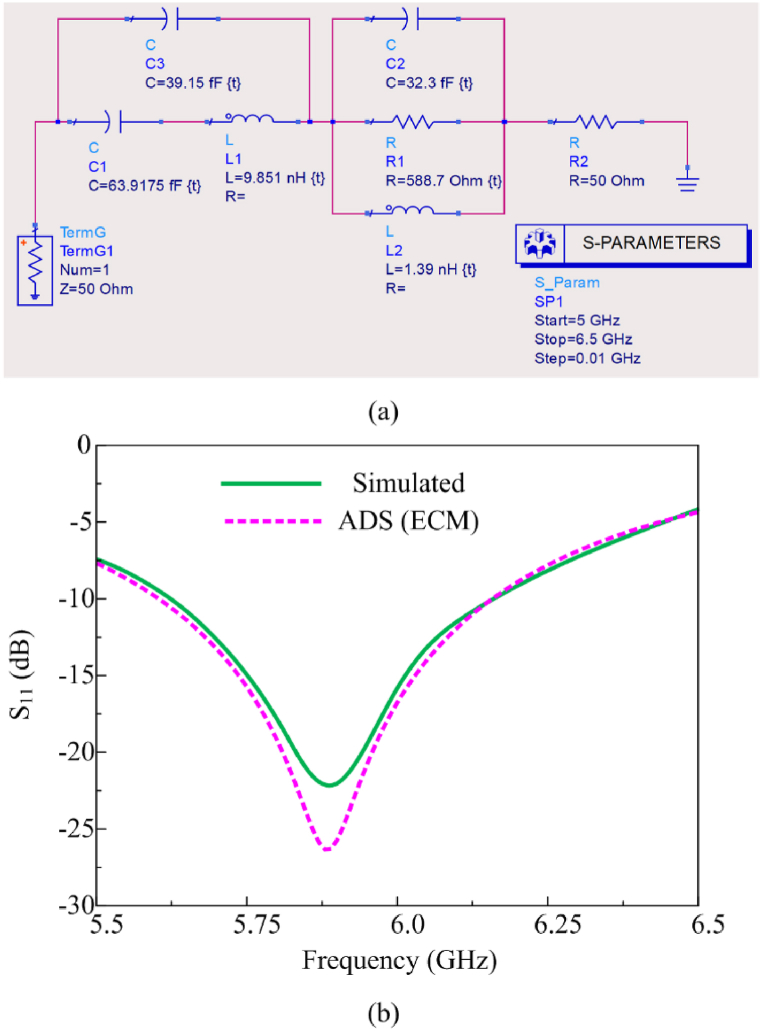
(a) Equivalent circuit model (ECM) of the proposed antenna (b) comparison among CST and ADS S_11_ results.

The equivalent circuit model of the proposed conformal antenna is depicted in Fig. 3. The antenna can be represented as resistor-inductor-capacitor (RLC) model by carefully evaluating the values of these components based upon the antenna bandwidth and quality factor, that can be evaluated using equations (1), (2), (3). Initially, the center frequency (f_0_) of the antenna is estimated to be using the impedance bandwidth by the following relation:

(1) $$f_{0}=\frac{f_{high}-f_{low}}{2}=\frac{6.16-5.62}{2}=5.89\;GHz$$

Based on the central frequency the fractional bandwidth (F.B.) can be found with the help of the following expression:

(2) $$F.B\;\left(\%\right)=\frac{f_{high}-f_{low}}{f_{0}}*100=\frac{6.16-5.62}{5.89}*100=9.16\%$$

Finally, the quality factor of the antenna is estimated in terms of FBW, as both are inversely related to each other:

(3) $$Q_{F}=\frac{1}{F.B}=\frac{1}{0.0916}=10.91$$

Based upon the quality factor the RLC component of the proposed antenna can be estimated by utilizing equations (4), (5), (6), presented below.

(4) $$C_{2}=\frac{1}{2{\pi f}_{0}Z_{0}Q_{F}}$$

(5) $$L_{2}=\frac{1}{{\left(2\pi f_{0}\right)}^{2}C_{2}}$$

(6) $$R_{1}=Z_{0\;}\times Q_{F}$$

Here $Z_{0}$ represents the characteristics of the system which is Ω. By substituting the values of all the variable consecutively, equation gives the values approximated to C_2_ = 32.3 fF, L_2_ = 1.39 nH, and R_2_ = 588.7 Ω.

Moreover, the C1 and L1 show the equivalent circuit for the feed line. Although a well-matched circuit is estimated, still, the overall impedance offered by the circuit is 54.4 + j348.1 Ω. The following equations (7), (8), (9), (10), (11), (12) are utilized to estimate the overall impedance of the RLC circuit:

(7) $$Z_{C}=\frac{1}{j\omega C}$$

(8) $$Z_{L}=j\omega L$$

(9) $$Z_{R}=R$$

(10) $$Z_{1}=Z_{C1}+Z_{L1}$$

(11) $$Z_{2}=\frac{1}{Z_{R2}}+\frac{1}{Z_{L2}}+\frac{1}{Z_{C2}}$$

(12) $$Z_{Total}=Z_{1}+Z_{2}$$

The real part is close to the ideal value of the 50 Ω, however, the imaginary part shows the load is highly reactive. The comparison among CST and ADS results is presented in Fig. 3(b), offering a good comparison among both results. Thus, to minimize the reactive loading of the circuit a shunt capacitor of 39.15 fF is added along series connected C_1_ and L_1_, as shown in Fig. 3(a). After the addition of the shunt resistance the overall impedance of the circuit becomes 49.1 – j2.9 Ω, which is very close to the ideal value of 50 + j0 Ω. Thus, the proposed circuit can be used for the generation of similar results as offered by proposed antenna.

## 3 Multi-slotted wearable antenna results

**Fig. 4:**
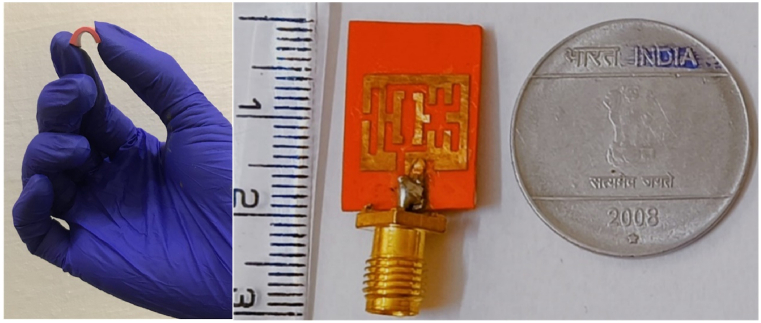
Fabricated Multi-slotted Wearable Antenna on a flexible silicon rubber substrate.

**Fig. 5:**
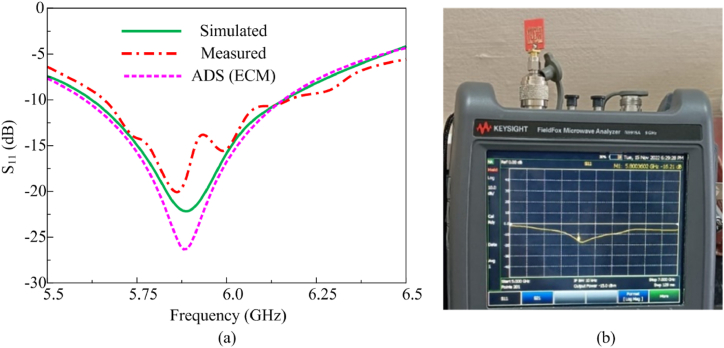
Multi-slotted wearable antenna (a) S_11_ measurement using Network Analyzer (b) simulated & measured S_11_ characteristics.

Additionally, the slot sizes are finalized by performing a parametric analysis concerning the length and width of the slots. The simulated antenna has an operating frequency range from 5.62 GHz to 6.16 GHz with the resonant frequency at 5.8 GHz. As per International Telecommunication Union (ITU) radio regulations, between 5.725 GHz and 5.875 GHz with a bandwidth of 150 MHz is considered an ISM unlicensed band used for medical, wearable, industrial, and scientific applications [44]. In the proposed design, a bandwidth of 540 MHz is achieved, which includes the ITU-recommended band of operation. Fig. 4 shows the fabricated antenna having a size of 14 mm × 12 mm x 2 mm. The comparison between s-parameter and the network characteristics of the fabricated antenna are tested using a vector network analyzer shown in Fig. 5. At 5.8 GHz, the minimum S11 obtained is −16.21 dB and the fabricated antenna very closely matches the simulated one. The fabricated one has a −10 dB operating band from 5.68 GHz to 6.17 GHz having a bandwidth of 490 MHz and the pictorial view is illustrated in Fig. 5(a and b).

**Fig. 6:**
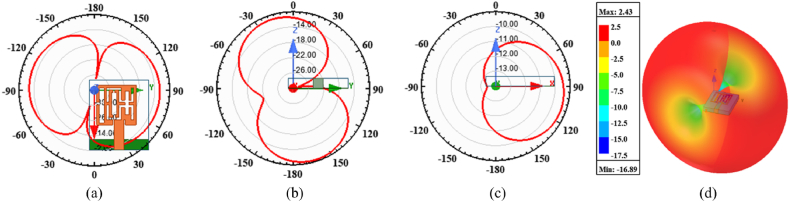
Multi-slotted Wearable Antenna performances at 5.8 GHz (a) XY plane pattern (b) YZ plane pattern (c) ZX plane pattern (d) 3D Gain pattern.

The suggested multi-slotted antenna's simulated voltage standing wave ratio (VSWR) is analyzed. The matching characteristics of an antenna are determined by the VSWR or Return Loss. It shows how effectively an antenna transmits and receives electromagnetic waves within a specific frequency range. The achieved VSWR for the operating frequency range is in the range of 1 and 2. The antenna gain depicts how well the designed antenna can radiate the radio waves in the desired direction according to the excited input signal. The suggested multi-slotted antenna radiation characteristics are analyzed in three different X-Y-Z planes and are shown in Fig. 6(a–c). The multi-slotted antenna radiating behavior is analyzed at 5.8 GHz center frequency. The multi-slotted antenna is placed along X-Y and Y-Z planes and radiates in a figure-of-8 pattern. In the X-Z plane, the antenna's energy radiates similarly to a broadside antenna radiating pattern. The three-dimensional (3D) antenna gain characteristics are shown in Fig. 6(d) at 5.8 GHz. A gain of 2.43 dBi is achieved. The designed antenna is linearly polarized.

**Fig. 7:**
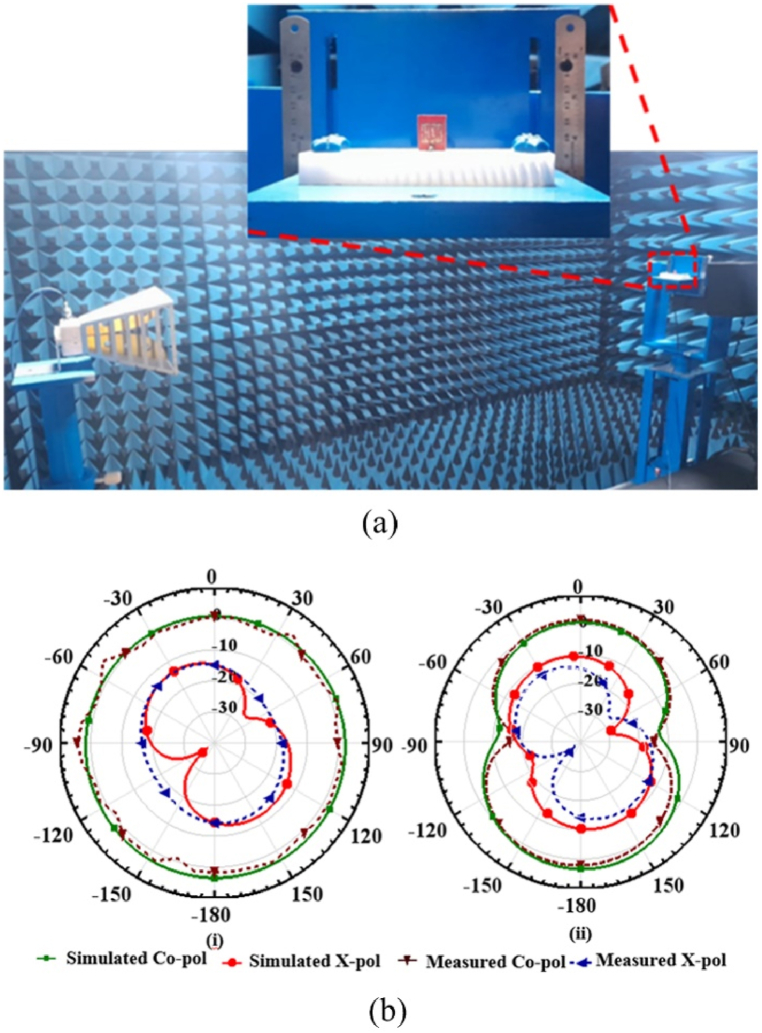
Multi-slotted Wearable Antenna Radiation Measurement at 5.8 GHz (a) Anechoic Chamber setup (b) (i) E-plane (ii) H-plane.

**Fig. 8:**
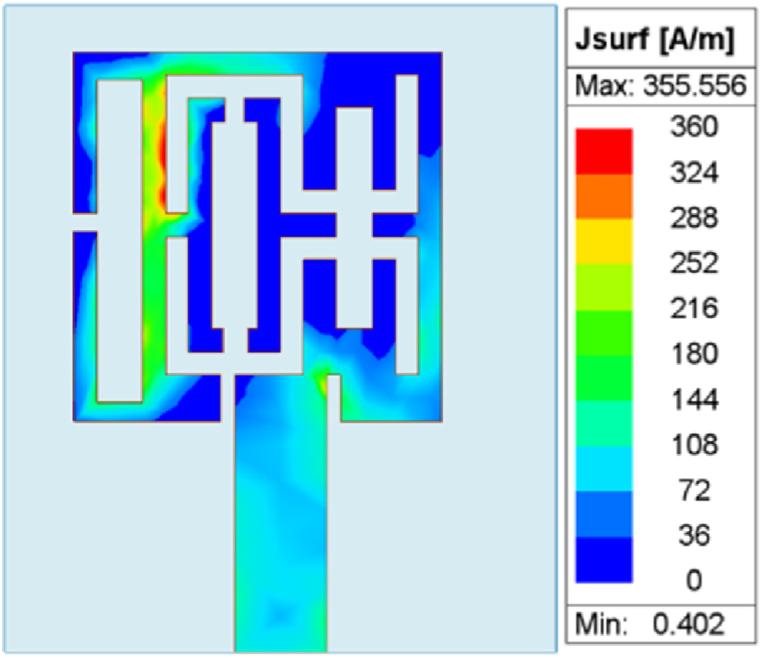
Multi-slotted wearable antenna current distribution at 5.8 GHz.

The anechoic chamber measurement setup and the radiation co-polarization measured characteristics in the E-plane and H-plane are depicted in Fig. 7 (a). The simulated co-polarization and cross-polarizations at 5.8 GHz are also depicted in the same measured characteristics. The simulation results show that there is sufficient difference in the co- and cross-polarization characteristics. For the multi-slotted antenna frequency range from 5.62 GHz to 6.16 GHz, the gain performance is between 2.36 dBi and 2.46 dBi, as depicted in Fig. 7 (b). The simulated directivity performance is also between 2.27 dBi and 2.33 dBi. The antenna radiation efficiency lies between 96 % and 98 % for the obtained band. Fig. 8 depicts the current distribution of the multi-slotted wearable antenna.

## 4 Multi-slotted wearable antenna – bending results

**Fig. 9:**
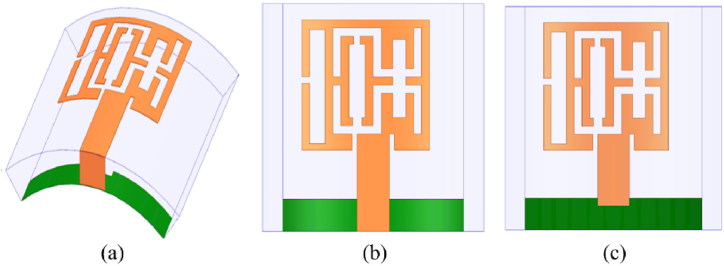
Multi-slotted Wearable Antenna Bending along the x-direction (a) Trimetric view, (b) Top view, (c) Bottom view.

**Fig. 10:**
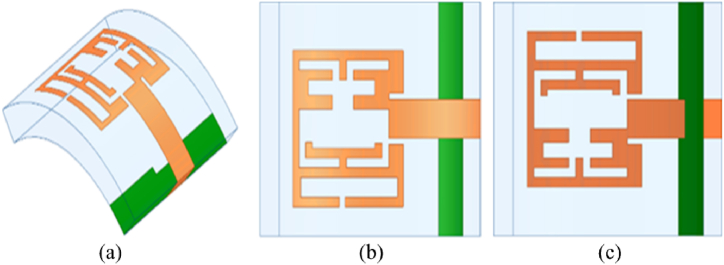
Multi-slotted Wearable Antenna Bending along the y-direction (a) Trimetric view, (b) Top view, (c) Bottom view.

**Fig. 11:**
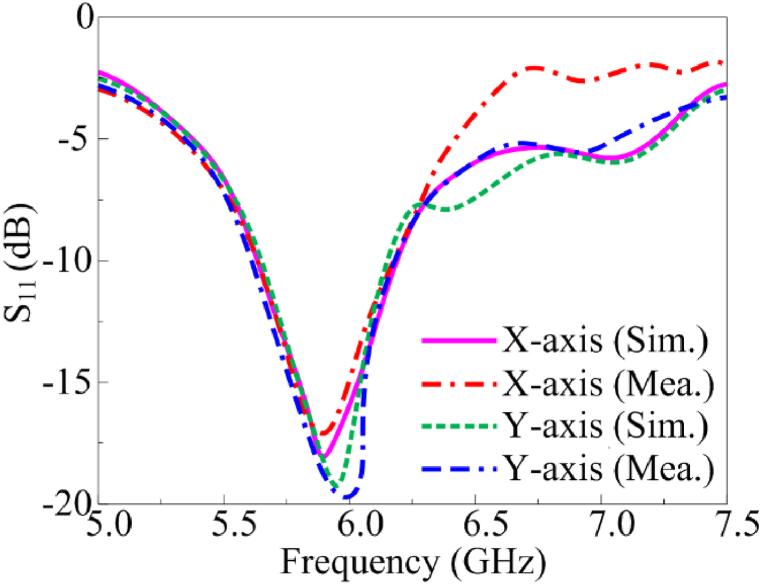
Multi-slotted wearable antenna bent S_11_ characteristics.

The proposed multi-slotted wearable antenna is designed by focusing on compact wearables like smartwatch applications. Once this type of device is worn in the hand, the antenna will undergo certain level of conformability. The antenna performance should remain constant or very close results should be obtained as of the antenna in a normal environment without facing any bending scenarios. Therefore, the proposed antenna is bent along x- and y-axis. The antenna is bent along the x-direction with an angle of 43.08° and a bending radius of 9 mm. Since the antenna is used for wearable hand applications, the bending radius considered is less. Fig. 9 (a) provides the bent structure of the multi-slotted antenna in a trimetric view, the top and bottom views are shown in Fig. 9 (b) and 9 (c), respectively. Similarly, Fig. 10(a–c) presented the proposed antenna under conformal conditions along the y-axis, where similar bending angle and radius are utilized as used for x-axis. The reflection characteristics of the bent antenna in both axes closely match the simulated antenna without any bending. The proposed antenna operates from 5.76 GHz to 6.26 GHz when bent along both axis and measured results are also similar to the simulated results, as depicted in Fig. 11.

**Fig. 12:**
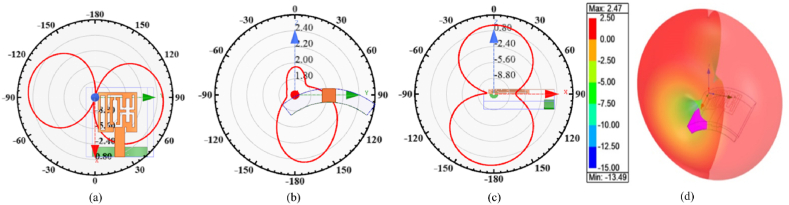
T Multi-slotted Wearable Antenna bending performances at 5.8 GHz (a) XY plane pattern (b) YZ plane pattern (c) ZX plane pattern (d) 3D Gain pattern.

**Fig. 13:**
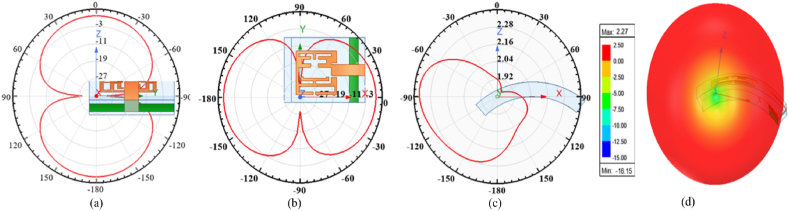
T Multi-slotted Wearable Antenna bending performances at 5.8 GHz (a) XY plane pattern (b) YZ plane pattern (c) ZX plane pattern (d) 3D Gain pattern.

The bent multi-slotted antenna radiation characteristics are analyzed in three different X-Y-Z planes when bent along x- and y-axis, as shown in Fig. 12(a–d) and Fig. 13(a–d). The multi-slotted antenna radiating behavior is analyzed at the central frequency of 5.8 GHz. When bent along x-axis the antenna offers dual beam radiation patterns in XY- and XZ-planes while a tilted omni-directional radiation pattern is observed in XZ-plane, consequently a smooth pattern observed in 3D plane, as depicted in Fig. 12. Similarly, the antenna offers dual beam patterns in XY- and YZ-plane while a directional pattern is observed in XZ-plane, as depicted in Fig. 13. The peak gain of 2.47 is observed for X-axis while for conformal analysis along Y-axis it show that the antenna has a maximum gain of 2.27. Thus, the antenna offers a more stable performance in X-axis as compared to bending along Y-axis.

## 5 Multi-slotted wearable antenna – User's left-hand analysis

**Fig. 14:**
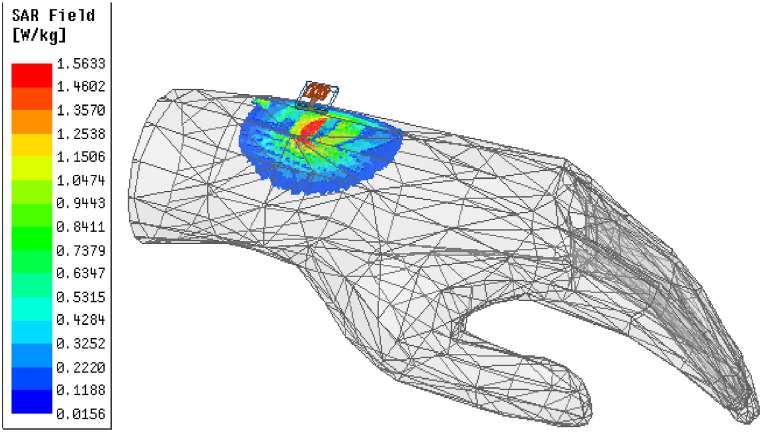
Multi-slotted wearable antenna user's left-hand SAR analysis.

**Fig. 15:**
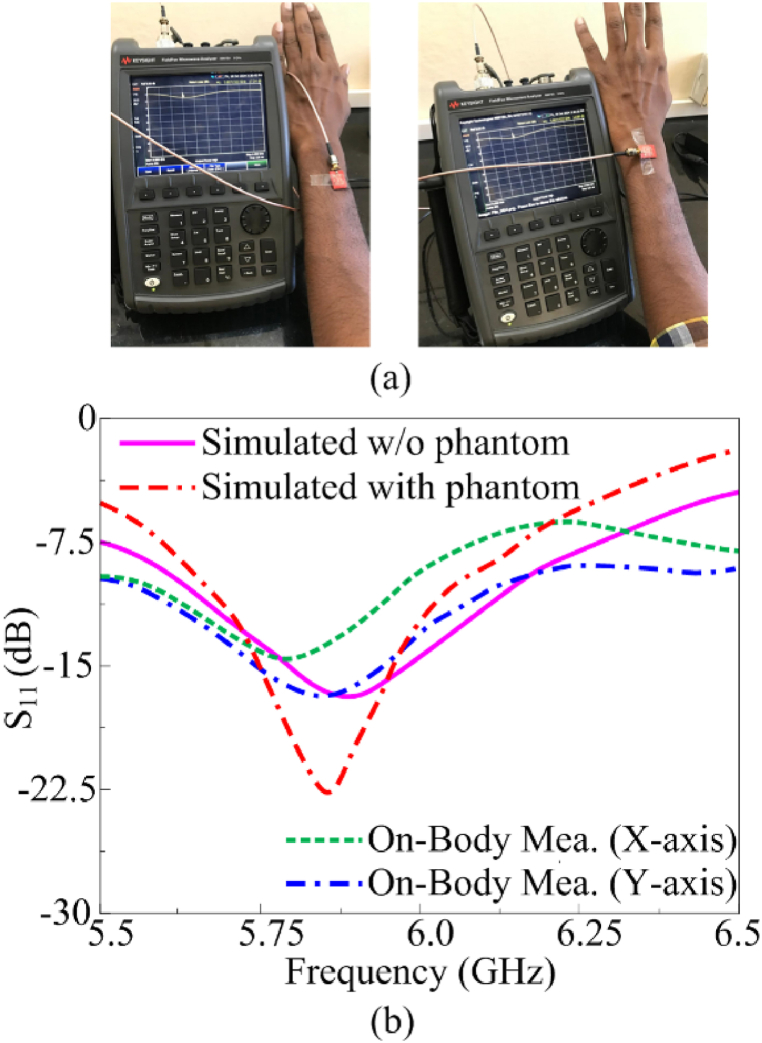
Multi-slotted wearable antenna user's left hand S_11_ characteristics.

Since the suggested multi-slotted antenna is used for body wearable applications, the specific absorption rate (SAR) of the antenna is analyzed in this section. By placing the antenna in the human hand as depicted in Fig. 14, the designed multi-slotted antenna shows a similar operating band and resonant frequency of operation as that of the antenna without the human hand. The antenna is placed 10 mm above the human hand. With phantom, the operating frequency range is from 5.66 GHz to 6.07 GHz having minimum S_11_ = −20 dB at 5.8 GHz, the measured setup is shown in Fig. 15(a) while s-parameters are shown in Fig. 15(b).

To validate the hand radiation absorption characteristics, a specific absorption rate (SAR) for 1 g of tissue is analyzed. SAR is a measurement of the quantity of RF energy that is transmitted and captivated by human tissue. The electrical conductivity, which is measured in Siemens per meter, the induced E-field from the radiated energy, which is measured in volts per meter, and the tissue mass density, which is measured in kg per cubic meter, all affect SAR. By averaging (or integrating) across a predetermined volume, usually a 1-g or 10-g area, the SAR is derived. The antenna is placed at a 10 mm distance from the user's left hand and an input power of 1W is used to excite the multi-slotted antenna. The SAR obtained is 1.56 W/kg for 1 g of tissue for the suggested multi-slotted antenna, as shown in Fig. 14.

**Fig. 16:**
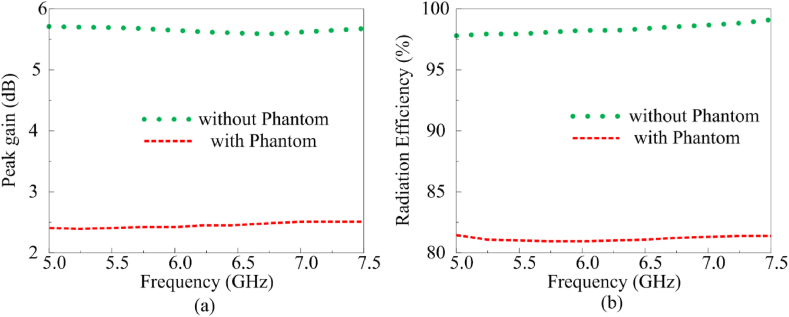
Multi-slotted wearable antenna (a) gain characteristics (b) radiation efficiency characteristics.

**Fig. 17:**
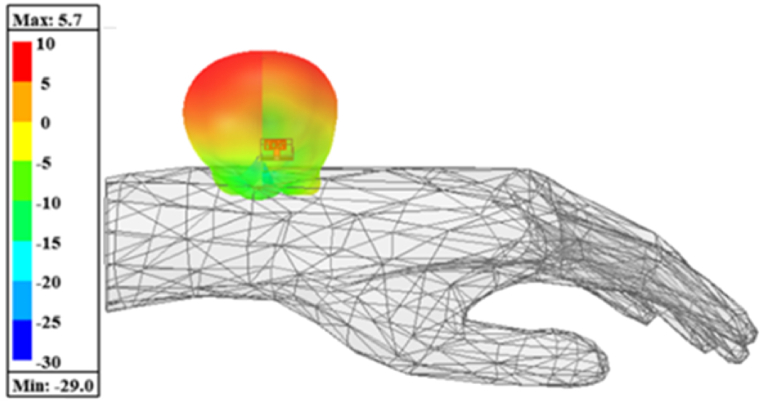
Multi-slotted wearable antenna 3D gain pattern with hand-model phantom.

The gain and radiation efficiency of the multi-slotted antenna is analyzed in free space and with the user's left-hand phantom, as shown in Fig. 16(a and b). The antenna peak increases from 2.46 dBi to 5.8 dBi due to backward radiation from the hand. Moreover, Fig. 17 depicts the broadside radiation characteristics of the antenna when loaded at the top of the hand phantom. Fig. 16 (b) depicts the radiation efficiency of the proposed antenna in free space and when loaded on top of hand model. The radiation decreases from 96 % to 80 % to the EM absorption caused by hand phantom, this also emphasis on studying the SAR characteristics of the antenna which are already discussed in previous section.

## 6 Comparison with literature work

The proposed flexible antenna is compared with recently reported conformal or semi-conformal antennas for fair comparison. The proposed work offers overperformance in all the related work by offering compact size, as shown in Table 1. Although the antenna reported in Refs. [47,48] offer wideband they either suffer from bigger dimensions or have limited flexibility, respectively. Moreover, the antenna proposed in Ref. [46] and [48 offers multi-band operation; however, their semi-flexible nature limits their usage for highly flexible devices like wearables. Thus, it can be concluded that proposed work offers a good combination of compact size, flexibility, broad bandwidth, and reasonable gain, making it a potential candidate for the on- and off-body applications.

**Table 1:** Comparative study with existing study of literature.

Year, Ref.	Antenna Size (λ_0_ x λ_0_)	Flexibility	Bandwidth (%)	Resonant Frequency (GHz)	Gain (dBi)	Max. SAR (W/kg)
2022 [25],	0.27 x 0.15	Flexible	0.03	2.45	2.17	0.98
2022 [45],	1 x 1.1	Flexible	0.028 | 0.03	2.45 | 5.8	5.3	0.49
2022 [46],	1.35 x 1	Semi-Flexible	0.03	5.8	>9	–
2023 [47],	0.95 x 0.8	Flexible	0.43 | 0.21 | 0.19	1.6 | 3.5 | 5.29	<2	1.0651
2023 [48],	0.3 x 0.39	Semi-Flexible	130 | 280 | 0.43	2.45 |3.5 | 5.8	>3	1.48
2020 [49],	0.37 x 0.04	Rigid	0.08	5.8	5.6	–
2022 [50],	0.54 x 0.45	Rigid	0.16	5.8	2.38	–
2024 [51],	0.58 x 0.96	Rigid	–	5.8	3.93	–
Proposed	0.27 x 0.23	Flexible	0.092	5.8	2.46	1.56

## 7 Conclusions

A potential candidate for wearable technology through the development and analysis of a multi-slotted antenna specially designed for use in wearable devices targeting wrist applications like smart watches. By utilizing a flexible and long-lasting silicone rubber substrate, this antenna effectively meets the increasing need for compact and versatile devices in the ever-evolving field of body area networks (BANs). The antenna's compact size of 14 mm × 12 mm and 2 mm substrate thickness make it ideal for wearable technology. With a resonance frequency of 5.8 GHz and a gain of 2.46 dBi, this antenna excels at transmitting and receiving signals over long distances. Most importantly, its specific absorption rate (SAR) of 1.56 W/kg per gram of tissue falls well within FCC safety standards, providing reassurance for extended use. The antenna offers high performance with a radiation efficiency of 96 % for free space and 80 % when working close to the human body. Moreover, an impedance bandwidth of 55 MHz is achieved ranges 5.66 GHz–6.07 GHz. Additionally, the bending characteristics analysis confirms that the antenna parameters align closely with the original flat analysis, which further emphasizes the design's flexibility and durability. The flexibility and durability of antennas make them adaptable for more than just wearable technology. They are also valuable in a range of industries, from military and medical to GPS, RFID, and fitness tracking.

## CRediT authorship contribution statement

**Kummaramsetty Sainath:** Writing – original draft, Methodology, Formal analysis, Conceptualization. **Shine Let Gunamony:** Writing – original draft, Software, Methodology, Investigation. **Wahaj Abbas Awan:** Writing – review & editing, Writing – original draft, Supervision, Software, Resources, Conceptualization. **Navin M. George:** Writing – original draft, Investigation, Formal analysis, Data curation. **Majjiga Deva Sindhu:** Writing – original draft, Validation, Resources, Methodology, Investigation. **Fahad N. Alsunaydih:** Writing – review & editing, Resources, Project administration, Funding acquisition. **Khaled Alhassoon:** Writing – review & editing, Supervision, Funding acquisition, Conceptualization.

## Data availability statement

Data included in article/supp. Material/referenced in article.

## Funding

The Researchers would like to thank the Deanship of Graduate Studies and Scientiﬁc Research at 10.13039/501100007414Qassim University for ﬁnancial support (QU-APC-2024-9/1).

## Declaration of competing interest

The authors declare that they have no known competing financial interests or personal relationships that could have appeared to influence the work reported in this paper.
